# Decomposing socioeconomic inequalities in dental caries among Chinese adults: findings from the 4th national oral health survey

**DOI:** 10.1186/s12903-023-03037-4

**Published:** 2023-06-08

**Authors:** Qing Chang, Menglin Cheng, Mengru Xu, Shuo Du, Xing Wang, Xiping Feng, Baojun Tai, Deyu Hu, Huancai Lin, Bo Wang, Chunxiao Wang, Shuguo Zheng, Xuenan Liu, Wensheng Rong, Weijian Wang, Yanmei Dong, Yan Si

**Affiliations:** 1 The Second Dental Center, National Engineering Laboratory for Digital and Material Technology of Stomatology, Beijing Key Laboratory of Digital Stomatology, Peking University School and Hospital of Stomatology, Beijing, P.R. of China; 2 Department of Stomatology, Beijing Friendship Hospital, Capital Medical University, Beijing, China; 3 Department of Preventive Dentistry, National Engineering Laboratory for Digital and Material Technology of Stomatology, Beijing Key Laboratory of Digital Stomatology, Peking University School and Hospital of Stomatology, Beijing, P.R. of China; 4 Chinese Stomatological Association, Beijing, P.R. of China; 5 Shanghai Ninth People’s Hospital, Shanghai Jiao Tong University School of Medicine, Shanghai, P.R. of China; 6 School & Hospital of Stomatology, Wuhan University, Wuhan, P.R. of China; 7 West China School of Stomatology, Sichuan University, Chengdu, P.R. of China; 8 Guanghua School of Stomatology, Hospital of Stomatology, Sun Yetsen University, Guangzhou, P.R. of China; 9 Chinese Center for Disease Control and Prevention, Beijing, P.R. of China; 10 Department of Cariology and Endodontology, National Engineering Laboratory for Digital and Material Technology of Stomatology, Beijing Key Laboratory of Digital Stomatology, Peking University School and Hospital of Stomatology, Beijing, P.R. of China

**Keywords:** Adults, Inequality, Dental caries, Concentration index, Socioeconomic disparities in health

## Abstract

**Objective:**

This cross-sectional study aimed to investigate socioeconomic inequalities in dental caries among adults (35 years and older) in China and explore the contributions of various factors to these inequalities.

**Methods:**

This study included 10,983 adults (3,674 aged 35–44 years, 3,769 aged 55–64 years and 3,540 aged 65–74 years) who participated in the 4th National Oral Health Survey (2015–2016) in China. Dental caries status was evaluated by the decayed, missing and filled teeth (DMFT) index. Concentration indices (CIs) were applied to quantify the different degrees of socioeconomic-related inequality in DMFT, decayed teeth with crown or root caries (DT), missing teeth due to caries or other reasons (MT), and filled teeth without any primary or secondary caries (FT) among adults of different age groups. Decomposition analyses were conducted to identify the determinants and their associations with inequalities in DMFT.

**Results:**

The significant negative CI indicated that DMFT for the total sample were concentrated among socioeconomically disadvantaged adults (CI = − 0.06; 95% confidence interval [CI], − 0.073 to − 0.047). The CIs for DMFT for adults aged 55–64 and 65–74 years were − 0.038 (95% CI, − 0.057 to − 0.018) and − 0.039 (95% CI, − 0.056 to − 0.023), respectively, while the CI for DMFT for adults aged 35–44 years was not statistically significant (CI = − 0.002; 95% CI, − 0.022 to 0.018). The concentration indices of DT were negative and concentrated in disadvantaged populations, while FT showed pro-rich inequalities in all age groups. Decomposition analyses showed that age, education level, toothbrushing frequency, income and type of insurance contributed substantially to socioeconomic inequalities, accounting for 47.9%, 29.9%, 24.5%,19.1%, and 15.3%, respectively.

**Conclusion:**

Dental caries was disproportionately concentrated among socioeconomically disadvantaged adults in China. The results of these decomposition analyses are informative for policy-makers attempting to develop targeted health policy recommendations to reduce dental caries inequalities in China.

## Introduction

Dental caries is highly prevalent worldwide and has substantial adverse effects on individuals, despite being largely preventable. Untreated caries affected 2.3 billion people worldwide in 2017 [1]. Dental caries has a significant negative impact on individuals’ quality of life and disproportionately affects socially disadvantaged individuals [2, 3]. Socioeconomic factors are crucial factors in caries development [4]. A systematic review based on 155 studies reported the relationship between socioeconomic status (SES) and caries experience and found that education, income, and occupation were significantly associated with a higher risk of having untreated caries lesions or any caries experience [5]. A social gradient also exists between socioeconomic status and the prevalence and severity of dental caries [6]. As expected, socioeconomic inequalities in dental caries are detected in most developed and developing countries, such as the UK, Germany, Mongolia, and Iran [7–11]. This issue of health disparities has raised more awareness in the public health community. Many studies document the existence of socioeconomic inequality in oral health for both children and adults between and within countries [12, 13]. Socioeconomic factors are vital oral health determinants, and the inequality of such factors is a formidable challenge for enhancing public oral health [14].

According to the Third National Oral Health Epidemiological Survey in China in 2005, the prevalence of dental caries is 88.1% in adults aged 35–44 years and 98.4% in adults aged 65–74 years [15]. The high prevalence of dental caries in China leads to a heavy economic burden. Socioeconomic inequalities in dental caries have also been observed in China. Tingting Zhang et al. identified socioeconomic disparities in dental caries among Chinese preschool children [16]. Analysis of Guangdong Province adult oral health survey data, reported by Yuandong Qin et al., showed greater inequalities in dental caries treatment than in caries experience [17]. Socioeconomic inequalities in dental caries may also widely exist among adults in China, not just among children and not just in one district like Guangdong Province. However, there is limited nationally-representative analysis from China, especially concerning SES and adults. The evidence of the impact of SES on dental caries among adults in China is insufficient, and additional study is needed.

Quantification and measurement of inequality in oral health were obtained through the concentration index (CI). Plotting the concentration curve would represent inequality visually and decomposing the concentration index would identify the factors contributing to inequality [18, 19]. In the current study, the CI was employed to investigate socioeconomic inequalities in dental caries measured by the decayed, missing, and filled teeth (DMFT) index among adults (35 years and older) in China. Decomposition analysis was later performed to specify potential factors affecting socioeconomic inequality in dental caries. Measuring the degree of inequality in dental caries and investigating the factors contributing to inequality are important for informing equity-oriented health policies and practices. Therefore, this cross-sectional study aimed to examine socioeconomic inequalities in dental caries among adults (35 years and older) in China by computing concentration indices and investigating the contribution of various factors to this inequality through the decomposition approach.

## Method

### Data source

This study was analyzed based on data from the Fourth National Oral Health Survey (2015). The survey included all 31 provinces, autonomous regions, and municipalities in mainland China, using a multistage, stratified, random, equal proportion sampling method. Referring to the 3rd National Oral Health Survey (2005), periodontal disease among adults was 86%. The two-sided 95% confidence interval was set at 10%, and the design effect was 4.5. By considering the stratification factor and a non-response rate of 20%, the sample size was 4230 each for three age groups, including the 35 to 44, 55 to 64, and 65 to 74 age groups. Two urban and two rural districts were randomly selected in each province by probability proportional to size (PPS) sampling. Then three neighborhood communities and three villages were chosen using PPS sampling from each urban and rural district previously selected. Finally, 36 local residents were recruited by quota sampling in each neighborhood or village community. More detailed sampling methodologies have been described in previous articles [20, 21]. Finally, 4410 35- to 44-year-olds, 4623 55- to 64-year-olds, and 4431 65- to 74-year-olds completed the survey. After excluding the subjects with missing values of relevant variables, a total of 10 983 adults (3674 aged 35–44 years, 3769 aged 55–64 years, and 3540 aged 65–74 years) accounting for 81.6% of participants were included in this study.

Post-stratification weights were computed based on the participants’ province, residence (urban or rural area), age, and gender, according to the Sixth National Demographic Census in China, which was obtained online from the National Bureau of Statistics [22]. The study was approved by the Ethics Committee of the Chinese Stomatological Association, Beijing (No. 2014-003). After being provided with the details of the survey, all participants signed informed consent forms.

### Data collection

The collected data contained oral examinations and structured questionnaires at the survey site. Dental caries was examined by trained and accredited dentists using plane dental mirrors and Community Periodontal Index (CPI) probes under artificial light and assessed in the form of the DMFT index according to the WHO criteria [23]. The dental caries examinations exhibited inter- and intra-examiner kappa values higher than 0.8. To answer the questionnaires, participants were interviewed face-to-face by two or three trained and certified interviewers. The structured questionnaire was revised after the pilot study solicited information on demographic variables, education level, number of family members, household income, oral health promoting behaviors and insurance types.

### Variables

#### Oral health outcomes

DT, MT, and FT were employed to assess dental caries status. The DMFT index was calculated to describe the dental caries experience.

#### Annual household income per capita

The survey contained a question to gather participants’ annual household income information: “What was your approximate total household income in the past 12 months?” The answer required a multiple of ¥10,000. Furthermore, the number of family members was recorded.

#### Education

Education was classified into three levels: low (primary school or less), moderate (junior high school), and high (high school or above).

#### Oral health behavior

Consumption of desserts and sugared beverages was classified into three levels: rarely, one to three times a month and more than once a week. Toothbrushing frequency was classified into less than once a day, once a day and twice a day or more.

#### Sociodemographic variables

Sociodemographic variables included gender (male or female), age, nationality, occupation (agriculture, nonagriculture, unemployment, retirement), residential location (rural/urban) and region (east, middle, west).

#### Insurance types

There are three major types of basic medical insurance for citizens in China: Urban Employee Basic Medical Insurance (UEBMI) for the employees living in the urban, Urban Resident Basic Medical Insurance (URBMI) covering urban residents without contractual jobs or unemployed and the New Rural Cooperative Medical System (NCMS) for rural residents [24].

#### Health condition variables

Health condition variables included chronic disease (yes/no) and smoking (yes/no).

### Data analysis

Concentration curves and the CI were used to quantify socioeconomic inequalities in dental caries among Chinese adults. The concentration curve graphs the cumulative proportion of health outcome variables (DMFT, DT, MT, FT score) on the y-axis and the cumulative percentage of the population ranked by income on the x-axis [18]. If each member of society, irrespective of their socioeconomic status, has exactly the same health outcome, the concentration curve will be an equality line (45-degree line), and the value of CI will be zero. The CI is defined as twice the area between the concentration curve and the equality line, ranging from − 1 to 1 [25]. If the concentration curve lies below (above) the equality line, the sign of CI is positive (negative), indicating that the health variable is disproportionately concentrated among the rich (poor) [26]. The farther the curve is above/below the equality line, the greater the health variable is concentrated among the poor/rich.

The observed socioeconomic inequalities can be explained through the decomposition analysis of the CI, which identifies the contributions of various socioeconomic factors to the inequalities and examines the degree of socioeconomic inequality in that factor [18]. The decomposition analysis helped to reveal the linear associations between the health variable and covariates rather than a direction of causality. DMFT served as an outcome variable. Moreover, the explanatory variables included demographic variables, education level, income, oral health-related behaviors, insurance types, health conditions, residential location and region. The nature log of annual household income per capita was computed as the income variable. Each contribution was the product of the sensitivity of DMFT for that factor and the degree of socioeconomic-related inequality. The contribution of each factor can be either positive or negative. A positive value of contribution means that the variable would increase inequality in the health outcome variable (DMFT) and vice versa. The robustness of the decomposition analyses was assessed by sensitivity analyses. For each sensitivity analysis, any changes in the rank order of the contribution of determinants were assessed.

All data analyses were conducted by STATA SE 15.0. The guide to health equity analysis provided the decomposition analysis code.

## Results

Descriptive statistics of the study sample are presented in Table 1. The participants were 50.98% male and 49.02% female from eastern (32.81%), western (26.9%), and central (40.29%) China. The respondents were equally distributed across urban and rural areas. A total of 36.78% of individuals brushed their teeth twice a day or more, and 50.08% brushed their teeth once a day. Nearly half of the participants were enrolled in NRCMI, while 34.13% were in UEBMI and 12.27% were in URBMI. The fractions of individuals gaining a high school or higher education diploma were 40.94%, 22.63%, and 12.2% among adults aged 35–44 years, 55–64 years, and 65–74 years, respectively. A total of 43.14% of adults aged 35–44, 31.18% of adults aged 55–64, and 30.28% of adults aged 65–74 had sweets once a week or more.

**Table 1:** Characteristics of the study population by age group

Variables	35–44 y old		55–64 y old		65–74 y old		Total sample	
	n(N = 3674)	%	n(N = 3769)	%	n(N = 3540)	%	n(N = 10,983)	%
**Gender**								
Male	1853	50.44	1918	50.89	1828	51.64	5599	50.98
Female	1821	49.56	1851	49.11	1712	48.36	5384	49.02
**Regions**								
East	1202	32.72	1260	33.43	1142	32.26	3604	32.81
Middle	979	26.65	1011	26.82	964	27.23	2954	26.9
West	1493	40.64	1498	39.75	1434	40.51	4425	40.29
**Urban or rural area**								
Urban area	1851	50.38	1926	51.10	1841	52.01	5618	51.15
Rural area	1823	49.62	1843	48.90	1699	47.99	5365	48.85
**Education**								
Primary school or lower	793	21.58	1634	43.35	2049	57.88	4476	40.75
Middle school	1377	37.48	1282	34.01	1059	29.92	3718	33.85
High school or higher	1504	40.94	853	22.63	432	12.20	2789	25.39
**Basic Medical Insurance**								
UEBMI	1301	35.41	1275	33.83	1172	33.11	3748	34.13
URBMI	422	11.49	461	12.23	465	13.14	1348	12.27
NRCMI	1789	48.69	1911	50.7	1745	49.29	5445	49.58
**Annual household income per capita (Yuan)** ^**a**^	15783.29 (16385.83)		14047.88 (17118.24)		13878.76 (14822.69)		14573.90 (16182.87)	
**Sweet consumption**								
Never or rarely	899	24.47	1379	36.59	1418	40.06	3696	33.65
1–3 times a month	1190	32.39	1215	32.24	1050	29.66	3455	31.46
Once a week or more	1585	43.14	1175	31.18	1072	30.28	3832	34.89
**Toothbrushing Frequency**								
Less than once a day	252	6.86	535	14.19	656	18.53	1443	13.14
Once a day	1670	45.45	2055	54.52	1775	50.14	5500	50.08
Twice a day or more	1752	47.69	1179	31.28	1109	31.33	4040	36.78
**Smoking**								
Yes	1025	27.90	1067	28.31	857	24.21	2949	26.85
No	2649	72.10	2702	71.69	2683	75.79	8034	73.15
**Chronic disease**								
No	3120	84.43	1880	49.88	1327	37.49	6309	57.44
Yes	572	15.57	1889	50.12	2213	62.51	4674	42.56
**Nationality**								
Han	3286	89.44	3449	91.51	3226	91.13	9961	90.69
Minority	388	10.56	320	8.49	314	8.87	1022	9.31

^a^ demonstrated in mean (standard deviation) for annual household income per capita

Ninety-four percent of all participants had dental caries experience (DMFT ≥ 1). 63% of participants suffered from untreated caries (DT ≥ 1), and the prevalence rate for MT ≥ 1 was 86.23%. However, only 21.74% of participants received caries restoration. The basic dental caries characteristics of participants in this study are shown in Table 2. The mean (standard deviation [SD]) scores of D, M, F and DMFT of the total sample (adults aged 35–74 years) in this study were 2.39 (3.41), 5.75 (6.86), 0.52 (1.41) and 8.66 (7.92), respectively. The average scores of DT, MT, and DMFT increased with age.

**Table 2:** Mean (SD) scores of D, M, F and DMFT

	DT (SD)	MT (SD)	FT (SD)	DMFT (SD)
35–44 y	1.57(2.51)	2.39(2.32)	0.56(1.42)	4.52(3.95)
55–64 y	2.39(3.24)	5.63(6.14)	0.50(1.38)	8.52(7.12)
65–74 y	3.24(4.12)	9.37(8.69)	0.50(1.42)	13.11(9.32)
Total sample	2.39(3.41)	5.75(6.86)	0.52(1.41)	8.66(7.92)

**Fig. 1:**
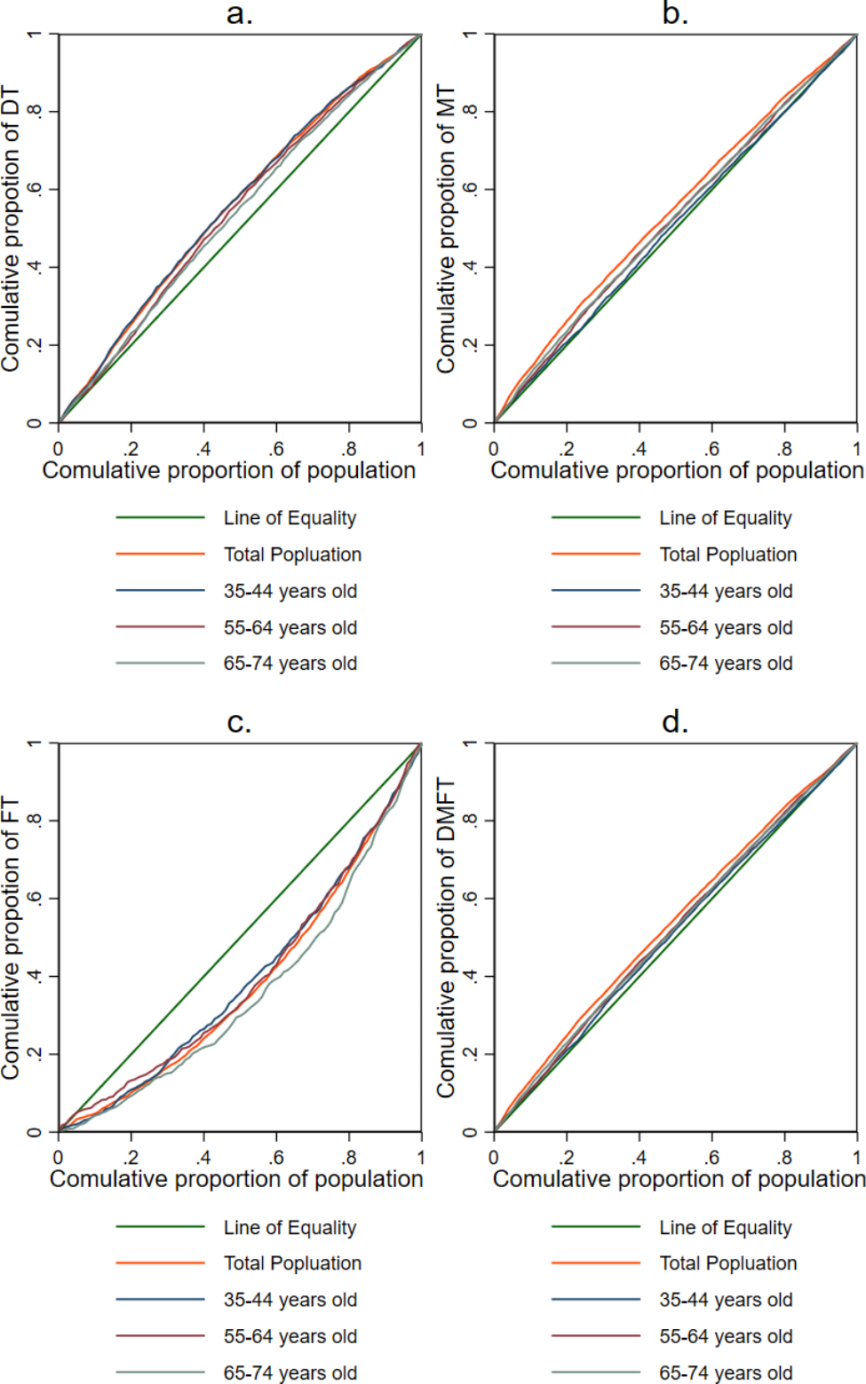
Concentration curves of DT, MT, FT, and DMFT. **a** Concentration curves of DT. **b** Concentration curves of MT. **c** Concentration curves of FT. **d** Concentration curves of DMFT.

Table 3 displays the CIs of DMFT, DT, MT, and FT of the total sample for each age group. The results showed that the DMFT of the total sample was concentrated in the disadvantaged population. The CI for DMFT for adults aged 35–44 years was not statistically significant, and the concentration curve (Fig. 1) was close to the perfect equal line, indicating that the distribution of DMFT was nearly equal. The CIs for DMFT for adults aged 55–64 and 65–74 years were similar and significantly negative, suggesting that DMFT were concentrated among poor individuals.

**Table 3:** Concentration indices of DMFT, DT, MT and FT

	DMFT (95% CI)	DT (95% CI)	MT (95% CI)	FT (95% CI)
35–44 y	-0.002	-0.103^***^	-0.016	0.202^***^
	(-0.022, 0.018)	(-0.139, -0.068)	(-0.006, 0.038)	(0.146, 0.258)
55–64 y	-0.038^***^	-0.100^***^	-0.038^***^	0.255^***^
	(-0.057, -0.018)	(-0.131, -0.688)	(-0.064, -0.011)	(0.187, 0.322)
65–74 y	-0.039^***^	-0.088^***^	-0.042^***^	0.372^***^
	(-0.056, -0.023)	(-0.117, -0.058)	(-0.064, -0.020)	(0.301, 0.443)
Total sample	-0.060^***^	-0.123^***^	-0.065^***^	0.249^***^
	(-0.073, -0.047)	(-0.142, -0.103)	(-0.082, -0.047)	(0.210, 0.288)

***P < 0.001

As depicted in Fig. 1, the concentration curves for DT for all age groups were located above the perfect equal line, indicating a disproportionate concentration of untreated dental caries among disadvantaged adults. Additionally, the concentration curve for FT was below the perfect line, which showed the pro-rich inequality. At the same time, the CI for FT for adults aged 65–74 years was higher than in other age groups, indicating greater inequality in that age group. The CI for MT for adults aged 35–44 was not statistically significant, whereas the CI for other age groups was significantly negative, suggesting pro-poor inequality (Table 2).

Table 4 displays the detailed results of the decomposition analyses of socioeconomic inequalities in DMFT across different age groups. For adults aged 35–74 years, age was the largest contributor, reflecting the unequal distribution of the DMFT index between individuals across different age groups. The other driving factors included educational achievement (29.94%), frequency of teeth brushing (24.54%), income level (19.05%), and type of basic medical insurance (15.34%). To further explore the contribution of socioeconomic factors to inequality, the CI of DMFT for adults aged 55–64 and 65–74 years were decomposed separately. Education attainment, frequency of toothbrushing, and basic medical insurance type were the three most dominant contributors to socioeconomic inequality in DMFT among adults aged 55–64 and 65–74 years, with varying degrees for the two age groups. The contributors to inequality for the 55–64 age group rank as follows: frequency of teeth brushing, education attainment, and basic medical insurance type, which accounted for 40.28%, 40.06%, and 38.61% of the total inequality, respectively. The frequency of teeth brushing, accounting for 39% of the total inequality, contributed most to the inequality among adults aged 65–74 years. Moreover, education attainment and basic medical insurance type explained 20.53% and 24.48% of the variance, respectively. On the other hand, income contributed 18.93% of the total inequality among adults aged 55–64 years and 14.99% among adults aged 65–74 years. The frequency of sweet consumption had a negative contribution in all three age groups, indicating that it decreased pro-poor inequality for DMFT. The region had a negative contribution in both age groups (adults aged 55–64 and 65–74 years), while residential location (urban or rural) made a negative contribution (-25.69%) in adults aged 55–64 years but a positive contribution (1.93%) in older adults aged 65–74 years. According to the sensitivity analyses, the findings were robust.

**Table 4:** Decomposition of socioeconomic-related inequalities in DMFT by age group

	55–64 years old				65–74 years old				Total sample (35–74 years old)			
Variables	Elasticity	CIs	Contribution	%	Elasticity	CIs	Contribution	%	Elasticity	CIs	Contribution	%
**Location (Urban area** ^**ref**^ **)**				**-25.69**				**1.93**				**-7.07**
**Rural**	-0.1141	-0.0879	0.0100	-25.69	0.0082	-0.0932	-0.0008	1.93	-0.0488	-0.0867	0.0042	-7.07
**Gender (Male** ^**ref**^ **)**				**4.06**				**1.76**				**3.82**
**Female**	0.1523	-0.0104	-0.0016	4.06	0.1171	-0.0059	-0.0007	1.76	0.2049	-0.0112	-0.0023	3.82
**Nationality (Han** ^**ref**^ **)**				**2.05**				**0.78**				**1.70**
**Minority**	0.0065	-0.1242	-0.0008	2.05	0.0085	-0.0359	-0.0003	0.78	0.0107	-0.0957	-0.0010	1.70
**Chronic disease (NO** ^**ref**^ **)**				**-0.16**				**-1.03**				**1.57**
**Yes**	0.0327	0.0020	0.0001	-0.16	0.0152	0.0266	0.0004	-1.03	0.0174	-0.0538	-0.0009	1.57
**Basic medical insurance (No insurance** ^**ref**^ **)**				**38.61**				**24.48**				**15.34**
**UEBMI**	-0.0702	0.4712	-0.0331	84.80	0.0108	0.5295	0.0057	-14.50	-0.0152	0.4174	-0.0064	10.64
**URBMI**	-0.0237	0.2038	-0.0048	12.38	0.0032	0.2294	0.0007	-1.89	-0.0078	0.1566	-0.0012	2.03
**NRCMI**	-0.0838	-0.2727	0.0228	-58.56	0.0530	-0.3027	-0.0161	40.87	0.0057	-0.2794	-0.0016	2.67
**Smoking (No** ^**ref**^ **)**				**0.34**				**2.95**				**0.42**
**Yes**	0.0301	-0.0044	-0.0001	0.34	0.0130	-0.0895	-0.0012	2.95	0.0149	-0.0168	-0.0003	0.42
**Sweet consumption (Rarely** ^**ref**^ **)**				**-18.14**				**-11.34**				**-13.79**
**1 to 3 times a month**	0.0098	-0.0164	-0.0002	0.41	0.0149	0.0024	0.0000	-0.09	0.0202	-0.0318	-0.0006	1.08
**Once a week or more**	0.0308	0.2346	0.0072	-18.55	0.0218	0.2023	0.0044	-11.25	0.0459	0.1937	0.0089	-14.87
**Toothbrushing (< 1/day** ^**ref**^ **)**				**40.06**				**39.21**				**24.54**
**Once a day**	-0.1633	-0.0367	0.0060	-15.35	-0.1315	-0.0256	0.0034	-8.58	-0.1877	-0.0816	0.0153	-25.58
**Twice a day or more**	-0.0847	0.2564	-0.0217	55.63	-0.0656	0.2861	-0.0188	47.79	-0.1404	0.2136	-0.0300	50.13
**Education (low** ^**ref**^ **)**				**40.06**				**20.53**				**29.94**
**Moderate**	-0.0336	0.0975	-0.0033	8.39	-0.0201	0.2127	-0.0043	0.1086	-0.0484	0.0120	-0.0006	0.97
**High**	-0.0402	0.3077	-0.0124	31.67	-0.0076	0.5028	-0.0038	9.67	-0.0500	0.3469	-0.0173	28.97
**Income**^**a**^	-0.0986	0.0749	-0.0074	**18.93**	-0.0652	0.0903	-0.0059	**14.99**	-0.1620	0.0704	-0.0114	**19.05**
**Region (East** ^**ref**^ **)**				**-22.61**				**-3.89**				**-12.34**
**Middle**	-0.0402	-0.1429	0.0057	-14.72	-0.0073	-0.1115	0.0008	-2.08	-0.0349	-0.1067	0.0037	-6.22
**West**	-0.0263	-0.1171	0.0031	-7.90	-0.0056	-0.1277	0.0007	-1.81	-0.0241	-0.1522	0.0037	-6.12
**Occupation (Agriculture** ^**ref**^ **)**				**14.40**				**9.42**				**3.58**
**Nonagriculture**	-0.0063	0.4008	-0.0025	6.52	-0.0033	0.4474	-0.0015	3.72	-0.0004	0.3325	-0.0001	0.20
**Unemployment**	-0.0102	0.0762	-0.0008	1.99	0.0121	0.0047	0.0001	-0.15	0.0035	-0.0022	0.0000	0.01
**Retirement**	-0.0058	0.3948	-0.0023	5.89	-0.0052	0.4407	-0.0023	5.84	-0.0060	0.3351	-0.0020	3.37
**Age group (35–44** ^**ref**^ **)**												**47.93**
**55–64**									0.1572	-0.0738	-0.0116	19.38
**65–74**									0.1804	-0.0947	-0.0171	28.55
**Sum**			-0.0359	92.13			-0.0402	99.79			-0.0686	114.72
**Residual term**			-0.0031	7.87			0.0009	0.21			0.0086	-14.72
**Total**			-0.0380	100.00			-0.0393	100.00			-0.0600	100.00

Aggregated contributions are in bold

Abbreviations: NRCMI, New Rural Cooperative Medical Scheme; UEBMI, Urban Employee Basic Medical Insurance; URBMI, Urban Resident Basic Medical Insurance

Ref, reference group

^a^ Natural log of annual household income per capita was calculated in the regression

## Discussion

In this cross-sectional analysis, the concentration index was utilized to investigate socioeconomic-related inequalities in dental caries and decomposition analysis identified the factors contributing to these inequalities. The results confirmed the presence of significant inequalities in oral health and found that DMFT in the over-35 population in China were concentrated among socioeconomically disadvantaged adults. Further decomposition analysis of the results showed that age, education level, teeth brushing frequency, income, and insurance type substantially contributed to the inequality. Previous studies suggested similar patterns of socioeconomic inequality in dental caries among adults in developed and developing countries [8, 13, 27]. Lower socioeconomic status is significantly associated with a greater risk of having any caries experience [5]. In addition, in our study, the CI for DMFT adults aged 55–74 years was negative, while that for adults aged 35–44 years was not statistically significant, suggesting that there is significant socioeconomic inequality in dental caries among adults aged 55 and over in China. A study by Liyan Wang et al. also found that middle-aged and older Chinese individuals with lower income and education levels were more likely to have dental caries [28].

Caries experience is a function of both caries prevalence and dental service utilization. The mean DMFT score in our study encompassed a larger portion of decayed teeth and missing teeth but a smaller portion of filled teeth. Moreover, DT was concentrated in disadvantaged populations, while FT showed a pro-rich inequality in all age groups. A previous study provided comparable results to this finding, where social gradients in untreated dental disease in children and adults were reported [29]. Disadvantaged individuals are more likely to show symptoms of dental pain and damaged teeth. In line with previous studies [17], our study indicated that people from a higher socioeconomic background receive more restorations than those of lower SES. A study conducted by XU found that dental service utilization was disproportionately concentrated in wealthier Chinese adults [30]. Lower dental service utilization might lead to accumulations of untreated dental conditions or advanced disease diagnosis. Additionally, the inequality of FT among people aged 65–74 was greater than in other groups. Treatment costs, mobility and transportation difficulties may add a substantial barrier to dental service utilization for older people [3].

After decomposing the CI of the total sample, it was observed that considerable inequality was attributable to age, which was a crucial factor in dentition and reflects the cumulative damage to oral health over time. The youngest and oldest groups were three generations apart, having very different lifetime exposures. This finding was consistent with the finding that significant socioeconomic inequality was associated with DMFT among adults aged 55–74 years, while the CI for DMFT among adults aged 35–44 years was not statistically significant. Previous studies also indicated that older individuals with low SES had a higher severity of DMFT [28]. The effects of the rest lifetime of inequality for the current generation of older people have been exposed. Considering the current aging process in the Chinese population, more attention in dental health care should be paid to seniors, especially those with disadvantaged backgrounds.

The decomposition results indicated that SES, such as household income and education level, was the primary determinant of socioeconomic-related inequality in DMFT among adults in China. A low income prevents people from accessing material benefits and services conducive to good health [31]. An unbalanced diet containing sugars and added fats, usually less expensive and more readily available in poor neighborhoods, can contribute to dental caries [2]. Cultural norms and educational background influence people’s health choices and behaviors [32]. In this study, education level played a prominent role in explaining inequalities in DMFT among adults in China. A series of studies have found that education consistently remains a vital contributor to inequalities in oral health, dental caries and dental service utilization [13, 17, 30]. Educational level frequently foreshadows the type of job, income, and general knowledge one could have [33]. Higher educational background generally raises awareness of access to preventive measures such as teeth cleaning habits, dental service utilization, and avoiding a high-carbohydrate diet [32, 34].

In addition to SES, toothbrushing frequency was a marked contributor to inequality in DMFT among adults in China. A systematic review found that people who brushed their teeth infrequently had a higher risk for caries than those who brushed frequently [35]. This suggests that dental caries may be largely determined by healthy and effective oral health care behaviors, such as oral hygiene habits and self-maintenance oral health. The study confirmed that education and toothbrushing frequency were significant determinants of socioeconomic inequality in DMFT among adults in China. The significance of good oral hygiene habits and oral health awareness cannot be overemphasized. While directly reducing social inequalities may be impossible, enhancing policies that directly raise dental awareness and knowledge of good oral health to promote oral health may be a more desirable solution.

In addition, this study suggested that the type of insurance was a marked contributor to the pro-poor inequality in dental caries. China has been trying to build up a universal public health insurance system for everyone by providing adequate health services at an affordable price. The Chinese government implemented a new round of health system reforms in 2009. There are three major public health insurance plans covering 90% of citizens in China. The NCMS covers rural residents, the UEBMI covers employees and retirees, and the URBMI provides coverage to children, students, seniors, and unemployed residents in urban areas. However, UEBMI and URBMI only cover basic dental health care costs, such as tooth extraction, caries restoration with amalgam or low-quality composite resin, and several other simple dental procedures. Over 85% of dental expenditures are not eligible for insurance reimbursement [36]. Insurance systems should help supply access to dental services for all residents and reduce health inequity. However, the results showed that the type of insurance affects the distribution of dental caries in the population. It contributed a positive percentage and increased inequality in dental caries. This may be related to the classification of the insurance system in China based on urban-rural status, which is essentially differentiated based on SES. Moreover, the limited insurance coverage of dental expenditures discourages people from seeking dental care services that can reduce socioeconomic inequalities in oral health. A previous study also found that the present basic medical insurance system in China contributed to socioeconomic-related inequality in dental utilization [30]. Thus, the Chinese health care insurance system has room for improvement, especially in dental care.

In recent years, China has begun to make an enormous amount of effort to achieve health equity including oral health, and has put forward a series of policies, such as the Healthy China 2030 Normative Outline (2016) and China’s Medium- and Long-Term Plan for the Prevention and Treatment of Chronic Diseases (2017–2030) (2017). The current study is the first to quantify and provide an overview of socioeconomic inequalities in dental caries among adults aged 35 and over in China. A concentration index and decomposition analysis were employed to comprehensively and systematically investigate socioeconomic inequalities in dental caries among Chinese adults. This study delved into the understanding of the relevant factors associated with dental caries inequality and provides evidence for further policymaking to reduce oral health inequality. In addition, the results of this study provide valuable information to form guidelines for evaluating current policies in China, such as the National Nutrition Program (2017–2030) and the Healthy China 2030 Plan, to assure better and more efficient future policies.

This study should be interpreted in terms of some limitations. First, since the study was conducted in a cross-sectional manner, causal relationships cannot be determined between explanatory variables and DMFT scores. Second, information on several related variables was obtained through self-reports or questionnaires, which could involve recall bias. Third, the DMFT score and its socioeconomic inequality can be influenced by more factors than the ones we have discussed.

## Conclusions

The results confirmed the presence of significant inequality in oral health and found that dental caries among the over-35 population in China was concentrated in socioeconomically disadvantaged adults. Further decomposition analysis of the results showed that age, education level, toothbrushing frequency, income and type of insurance substantially contributed to this pro-poor inequality. The results of decomposition analyses were informative for policymakers attempting to develop targeted health policy recommendations to reduce dental caries inequalities in China. Recommendations include implementing appropriate and targeted education programs for different socioeconomic groups and extending oral health promotion and education activities across the entire lifespan. Further attention should be given to oral health care for the elderly population, especially those who are poor.

## Data Availability

The data that support the findings of this study are available from the National Health Commission of the People’s Republic of China. Restrictions apply to the availability of these data, which were used under license for this study. Data are available from the authors with permission from the National Health Commission of the People’s Republic of China. The datasets used and analyzed in our study are available from corresponding author Yan Si.

## List of Abbreviations

DMFT: Decayed, missing and filled Teeth
DT: Decayed teeth with crown or root caries
MT: Missing teeth due to caries or other reasons
FT: Filled teeth without any primary or secondary caries
CI: Concentration index
SES: Socioeconomic status
PPS: Probability proportional to size
CPI: Community Periodontal Index
UEBMI: Urban Employee Basic Medical Insurance
URBMI: Urban Resident Basic Medical Insurance
NCMS: New Rural Cooperative Medical System
SD: Standard deviation
95% CI: 95% Confidence Interval
